# Dose-intense weekly cyclophosphamide, methotrexate, 5-fluorouracil, vincristine and prednisolone (CMFP) in advanced breast cancer.

**DOI:** 10.1038/bjc.1990.27

**Published:** 1990-01

**Authors:** J. S. Cebon, J. F. Bishop, V. Harvey, B. Mason, P. N. Jeal

**Affiliations:** Peter MacCallum Cancer Institute, Melbourne, Victoria, Australia.

## Abstract

Weekly chemotherapy with cyclophosphamide 80 mg m-2 day-1 p.o. continuously, methotrexate 35 mg m-2 week-1 i.v., 5-fluorouracil 500 mg m-2 week-1 i.v., vincristine 1.4 mg m-2 i.v. every two weeks and prednisolone 20 mg m-2 day-1 p.o. continuously (CMFVP) was prospectively studied in 45 previously untreated outpatients with advanced breast cancer to determine the feasibility of delivering a dose-intense regimen. Of 40 evaluable patients, complete response (CR) occurred in one patient, partial response (PR) in 20 (CR + PR 53%), stable in eight, progression in 11 and five were unevaluable for response. The median relapse-free survival for responders was 25 weeks and median survival for all patients was 31 weeks. The mean dose intensity relative to the Cooper regimen fell from 1.02 to 0.6 within the first 4 weeks of treatment and the median dose intensity achieved for all patients on study was only 0.52. Eighty-seven per cent of patients had treatment delays with a mean of 3.9 delays per patient and 71% had dose reductions. Neutropenia was the major toxicity with WHO grade 3 or 4 neutropenia (less than 1.0 x 10(9) l-1) in 62% of patients and three septic deaths while neutropenic. Dose-intense weekly CMFVP in this schedule cannot be delivered to previously untreated outpatients with advanced breast cancer.


*Br. J. Cancer* (1990), **61**, 133–136 © Macmillan Press Ltd., 1990

### Dose-intense weekly cyclophosphamide, methotrexate, 5-fluorouracil, vincristine and prednisolone (CMFP) in advanced breast cancer

J.S. Cebon[1], J.F. Bishop[1], V. Harvey[2], B. Mason[2] & P.N. Jeal[1]

[1]*Peter MacCallum Cancer Institute, 481 Little Lonsdale Street, Melbourne, Victoria 3000, Australia; and* [2]*Auckland Hospital, Auckland, New Zealand.*

**Summary** Weekly chemotherapy with cyclophosphamide 80 mg m$^{-2}$ day$^{-1}$ p.o. continuously, methotrexate 35 mg m$^{-2}$ week$^{-1}$ i.v., 5-fluorouracil 500 mg m$^{-2}$ week$^{-1}$ i.v., vincristine 1.4 mg m$^{-2}$ i.v. every two weeks and prednisolone 20 mg m$^{-2}$ day$^{-1}$ p.o. continuously (CMFVP) was prospectively studied in 45 previously untreated outpatients with advanced breast cancer to determine the feasibility of delivering a dose-intense regimen. Of 40 evaluable patients, complete response (CR) occurred in one patient, partial response (PR) in 20 (CR + PR 53%), stable in eight, progression in 11 and five were unevaluable for response. The median relapse-free survival for responders was 25 weeks and median survival for all patients was 31 weeks. The mean dose intensity relative to the Cooper regimen fell from 1.02 to 0.6 within the first 4 weeks of treatment and the median dose intensity achieved for all patients on study was only 0.52. Eighty-seven per cent of patients had treatment delays with a mean of 3.9 delays per patient and 71% had dose reductions. Neutropenia was the major toxicity with WHO grade 3 or 4 neutropenia ($<1.0 \times 10^9\,l^{-1}$) in 62% of patients and three septic deaths while neutropenic. Dose-intense weekly CMFVP in this schedule cannot be delivered to previously untreated outpatients with advanced breast cancer.

Chemotherapy for advanced breast cancer using the combination cyclophosphamide, methotrexate, 5-fluorouracil ± prednisolone CMF(P) given in a conventional intermittent schedule produces objective responses in approximately 50% of patients, with a response duration of 9 months (Smalley *et al.*, 1983; Tormey *et al.*, 1982). More recently attention has been directed towards the dose and schedule of therapy as a method of improving results with CMF in this disease.

The Cooper regimen, which included vincristine (CMFVP) and used a weekly schedule, appeared to be well tolerated with high responses in advanced disease and satisfactory results as adjuvant chemotherapy (Cooper, 1969; Cooper *et al.*, 1979). Hryniuk and Bush (1984) have studied the dose intensity of C, M and F in published data by converting each dose of drug to mg m$^{-2}$ week$^{-1}$ and comparing it directly to the Cooper regimen. They have concluded that many reported CMF programmes deliver much less intense chemotherapy than Cooper described and that a dose–response relationship may exist for C, M and F, especially if actual doses delivered are studied. Hrynuik and Bush's review was retrospective and not all published series of CMF(P) include dose delivery data.

To determine the feasibility of delivering a dose-intense regimen we studied a weekly CMFVP programme in previously untreated patients with advanced breast cancer. This programme was designed to deliver a dose intensity of 1.02 relative to the Cooper regimen.

### Materials and methods

*Patients*

Patients presenting to the Peter MacCallum Cancer Institute, Melbourne, Australia, or Auckland Hospital, New Zealand, with advanced metastatic breast cancer which was measurable or evaluable were eligible for this study. Eligible patients had ECOG performance status (PS) 0–3, had not received prior chemotherapy for advanced disease and a minimum of 6 months had elapsed since prior adjuvant chemotherapy. Prior, but not concurrent, radiotherapy and endocrine therapy were permitted.

Correspondence: J.F. Bishop
Received 17 November 1988; and in revised form 21 August 1989.

*Treatment plan*

Patients received cyclophosphamide 80 mg m$^{-2}$ day$^{-1}$ p.o. continuously (560 mg m$^{-2}$ week$^{-1}$), methotrexate 35 mg m$^{-2}$ i.v. weekly, 5-fluorouracil 500 mg m$^{-2}$ i.v. weekly, vincristine 1.4 mg m$^{-2}$ (maximum 2 mg) i.v. every 2 weeks and prednisolone 20 mg m$^{-2}$ day$^{-1}$ p.o. continuously (CMFVP). Prednisolone was used in this dose so that it could be given in equivalent dose intensity to the commonly used day 1 and day 8 intermittent CMFP. One course was defined as four successive weeks of treatment. Patients were seen and treated weekly. Prochlorperazine 12.5 mg i.v. or 25 mg by suppository or metoclopramide 10–20 mg i.v. were routinely given as anti-emetics.

Treatment was continued for at least 24 weeks unless disease progression occurred. Standard WHO response criteria were used (Miller *et al.*, 1981). Relapse-free survival was calculated for complete or partial responders only and was measured from the first day of treatment. Time to disease progression was calculated for all patients and measured from the first day of therapy.

All toxicity was prospectively recorded weekly using WHO toxicity criteria (Miller *et al.*, 1981). To assess this programme as an outpatient regimen, treatment was delayed if neutrophils were $<1.5 \times 10^9\,l^{-1}$ (WHO grade 2) or platelets $<75 \times 10^9\,l^{-1}$ (WHO grade 2) or if grade 2 or worse mucositis or other toxicity occurred. Doses of C, M and F were reduced by 25% if sepsis or bleeding occurred with WHO grade 2 toxicity or for WHO grade 3 or 4 toxicity (neutrophils $<1.0 \times 10^9\,l^{-1}$). Vincristine was omitted for severe neuropathy (WHO grade 3 or more).

*Calculation of dose intensity*

Dose intensity was calculated for each patient by converting the total dose of C, M, F and V into the dose received in mg m$^{-2}$ week$^{-1}$. The dose intensities for each drug were then expressed relative to the dose intensities of C, M, F or V in the Cooper regimen (Cooper, 1969; Cooper *et al.*, 1979) (cyclophosphamide 560 mg m$^{-2}$ week$^{-1}$, methotrexate 28 mg m$^{-2}$ week$^{-1}$, 5-fluorouracil 450 mg m$^{-2}$ week$^{-1}$), which was taken as 1.0. The average dose intensity (DI) was then calculated by averaging the DI of each drug and expressing it as a three drug (CMF) or four drug (CMFV) DI. The DI of the starting dose of the weekly programme outlined in the treatment plan was 1.02 relative to Cooper. However, prednisolone given in this programme is much more intense than in the Cooper regimen but not included in the calculation of dose intensity.

Patients who failed to complete one course of treatment (i.e. <4 weekly treatments) had a high calculated dose-intensity but clearly inadequate therapy. These patients were identified separately in our correlation of dose intensity with response. Dose intensity was calculated for each patient weekly. For each patient the profile of any change in dose intensity was determined and the complete dose intensity experience was called the cumulative dose intensity. The average cumulative dose intensity for all patients was calculated and graphed. The log rank test was used to compare survival curves which were generated using the Kaplan–Meier method (Peto *et al.*, 1977).

### Results

### *Patient characteristics*

Forty-five patients were treated with weekly CMFVP. The median age of patients was 55 years (range 31–79). On study entry, eight had ECOG PS 0, 14 had PS 1, 13 had PS 2 and 10 had PS 3. Only five patients received prior adjuvant chemotherapy and 37 had prior endocrine treatment. Sixteen patients received no prior radiotherapy and 29 had limited regional radiotherapy only. Only three patients had extensive radiotherapy to >50% of bone marrow areas.

### *Tumour response*

Five patients were inevaluable for response, two because of early septic death; two because they received two or less weekly doses and refused further treatment and one ceased therapy after one dose following a perforated peptic ulcer. All 45 patients are included on survival curves in calculations of dose intensity. Of 40 patients evaluable for response, one achieved a CR and 20 PR for an overall response (CR and PR) of 53%, eight had stable disease and 11 progressed on therapy. The median time to disease progression for all patients was 19 weeks, relapse-free survival (for CR + PR) was 25 weeks and median survival was 31 weeks.

### *Dose delivery*

For all 45 patients, 651 weeks on study were documented with 476 weekly treatments received. The first 27 patients (60%) received full dose initially. The subsequent 18 patients started 0.8 of the initial dose because of poor tolerance in the first 27 patients. No patients started at doses <0.8 of protocol doses. Seventy-one per cent of all patients had dose reductions of CMF on this program. A further 16% of reductions were vincristine alone.

Nine per cent of patients had vincristine related grade 2 or 3 neurotoxicity requiring dose reduction or omission, 5% had vincristine stopped with severe constipation and 2% had vincristine stopped for mild neurotoxicity outside protocol guidelines. Eighty-seven per cent of patients had one or more treatment delays with a mean number of delays per patient of 3.9 (Figure 1). In 47% of patients, treatment was stopped before relapse or progression by the physician because of toxicity and a further 16% stopped because of patient refusal.

**Figure 1** Treatment delays.

### *Toxicity*

The major toxicity encountered was haematological with WHO grade 3 or 4 neutropenia ($<1.0 \times 10^9 l^{-1}$) in 62 patients (Table I) and 38% of courses. Neutropenia was the main cause of dose reduction and treatment delay. There were three septic deaths associated with grade 4 neutropenia. Two patients perforated duodenal ulcers, one while on therapy and one patient within one month of completing therapy. WHO grade 2–4 mucositis occurred in 28% of patients. There were few gastrointestinal side-effects with WHO grade 2 or more nausea and vomiting in only 17% of patients.

### *Dose intensity*

The median four drug DI achieved on this study was only 0.52 and three drug DI was 0.53 (Figure 2). The cumulative mean four drug DI dropped from 1.02 at the start of therapy to 0.6 after only four weekly treatments (Figure 3).

### *Correlation of dose intensity and clinical outcome*

The numbers compared are small and no correlation could be detected between four drugs DI and clinical response (Figure 4). Since the median four drug DI was 0.52, patients were divided into two groups with DI <0.52 and >0.52. These groups were well balanced for pre-treatment characteristics such as age and performance status. There were no differences detected in time to disease progression or relapse-free survival for these two groups. The median survival of patients receiving DI >0.52 was 36 weeks and 30 weeks for DI <0.52 ($P = 0.8$). Similarly, there was no correlation detected between any of these clinical outcomes and three drug DI.

**Table I** Toxicity of patients receiving weekly CMFVP ($n = 45$)

| | WHO grade (%) | | | | |
|---|---|---|---|---|---|
| | *0* | *1* | *2* | *3* | *4* |
| Haemoglobin | 27 | 33 | 24 | 11 | 2 |
| Neutrophils | 11 | 18 | 7 | 38 | 24 |
| Platelets | 64 | 22 | 6 | 0 | 4 |
| Mucositis | 51 | 20 | 15 | 11 | 2 |
| Nausea and vomiting | 42 | 40 | 15 | 2 | 0 |
| Alopecia | 47 | 13 | 24 | 16 | 0 |
| Neurotoxicity | 58 | 33 | 7 | 2 | 0 |

**Figure 2** Dose intensity achieved.

**Figure 3** Cumulative mean four drug dose intensity.

**Figure 4** Correlation of dose intensity and response.

### Discussion

This weekly CMFVP programme produced objective responses in 53% of evaluable patients and 47% of all study patients. The median duration of objective response (CR or PR) was 25 weeks. These results are similar and not superior to published data with CMF(VP) given on intermittent schedules (Smalley *et al.*, 1976, 1983; Tormey *et al.*, 1982; Muss *et al.*, 1977). Cooper's results with weekly CMFVP are outstanding, but the intensity of the dose actually delivered on that programme has not been assessed (Cooper, 1969; Cooper *et al.*, 1979).

Patients entered on this study were not heavily pre-treated and would not be expected to have compromised bone marrow function. Only five had received prior adjuvant chemotherapy and had not received any chemotherapy for at least 6 months. Only three patients had received extensive prior radiotherapy while others received either no radiation or limited regional therapy only. Only four patients had proven or suspected bone marrow metastases. However, grade 3 or 4 neutropenia occurred in 62% of patients, with three septic deaths associated with grade 4 neutropenia even with the conservative policy of delaying therapy when neutrophils fell below $1.5 \times 10^9 l^{-1}$ and of dose reductions for grade 3 or 4 cytopenia.

Neutropenia was the major cause of dose reduction in 71% of patients. It also caused treatment delays in 87%. Most dose reductions or delays due to neutropenia occurred in the first 4 weeks of treatment with the mean cumulative dose intensity falling from 1.02 to 0.6 at four weeks (Figure 3). Thus, weekly treatments at this dose cannot be sustained and this was not due to prolonged chemotherapy with an increasingly compromised bone marrow. Following a delay, we attempted to recommence the same dose of chemotherapy as suggested by a mean of four treatment delays per patient. However, on average, only half of the intended dose intensity could be finally delivered even in patients who had 0.8 of the starting dose. Thus, it is unlikely that weekly CMFVP can be applied intensively without a commitment to prolonged hospitalisation in most patients.

The programme was otherwise well tolerated with little gastrointestinal toxicity. In 16% of patients, vincristine had to be withdrawn because of neurotoxicity. Weekly therapy doubled the size of an already large outpatient clinic and resulted in seven patients refusing further therapy.

Similar continuous but less intense regimens have been randomly compared to intermittent CMFVP and achieved improved responses but no differences in survival (Smalley *et al.*, 1976; Muss *et al.*, 1977). Increased dose intensity of doxorubicin has resulted in increased objective responses (Jones *et al.*, 1987). Hyrniuk and Bush (1984) in a retrospective analysis suggested that improvements in the treatment of advanced breast cancer may result from delivery of more dose intense therapy. Patients randomised to dose escalation and protected environment using chemotherapy with 5-fluorouracil, doxorubicin and cyclophosphamide (FAC) failed to demonstrate an advantage for more intense therapy (Hortobagyi *et al.*, 1987). However, in that study a relatively narrow dose intensity range was achieved

In this prospective study, no clear correlation or trend for improved response, time to progression, relapse-free survival or survival could be detected for patients with higher dose intensity. However, the number of patients in whom we have made clinical correlations with dose intensity are small and the range of dose intensity is quite small so that firm conclusions regarding the value of increased dose intensity cannot be made. A randomised study design comparing two regimens with substantially different dose intensity is required to resolve this issue.

High dose prednisolone, when added to standard intermittent CMFP, significantly improved response duration and survival in a randomised trial (Tormey *et al.*, 1982). The combination incorporating high dose prednisolone, CMFP, has been widely used in Australia and was shown to be equivalent to adriamycin and cyclophosphamide in previously untreated patients (Coates *et al.*, 1987). In addition, we planned this phase II study with the same dose intensity of prednisolone so that it could be compared with standard intermittent CMFP in subsequent studies. However, the prednisolone may have contributed to the toxicity in two patients who perforated duodenal ulcers.

We were unable to deliver dose-intense therapy to outpatients with this weekly schedule. Other methods of dose intensification such as high dose, intermittent schedules, with bone marrow rescue or recombinant colony stimulating factors may be more productive (Eder *et al.*, 1986).

### References

COATES, A., GEBSKI, V., BISHOP, J.F. *et al.* (1987). Improving quality of life during chemotherapy for advanced breast cancer. *N. Engl. J. Med.*, **28**, 1490.

COOPER, R.G. (1969). Combination chemotherapy in hormone resistant breast cancer. *Proc. Am. Assoc. Cancer Res.*, **10**, 15 (abstract).

COOPER, R.G., HOLLAND, J.F. & GLIDEWALL, O. (1979). Adjuvant chemotherapy of breast cancer. *Cancer*, **44**, 793.

EDER, J.P., ANTMAN, K., PETERS, W. & 8 others (1986). High dose combination alkylating agent chemotherapy with autologous bone marrow support for metastatic breast cancer. *J Clin. Oncol.*, **4**, 1592.

HORTOBAGYI, G., BUZDAR, AU., BODEY, G.P. & 5 others (1987). High dose inductor chemotherapy of metastatic breast cancer in protected environment: à prospective randomized study. *J. Clin. Oncol.*, **5**, 178.

HYRNIUK, W. & BUSH, H. (1984). Chemotherapy of metastatic breast cancer. *J. Clin. Oncol.*, **2**, 1281.

JONES, R.B., HOLLAND, J.F., BHARDWAJ, S., NORTON, L., WILFINGER, C. & STRASHUN, A. (1987). A phase I–II study of intensive-dose adriamycin for advanced breast cancer. *J. Clin. Oncol.*, **5**, 172.

MILLER, A.B., HOOGSTRATEN, B., STAQUET, M. & WINKLER, A. (1981). Reporting results of cancer treatment. *Cancer*, **47**, 207.

MUSS, H.B., WHITE, D.R. & COOPER, R. (1977). Combination chemotherapy in advanced breast cancer. *Ann. Intern. Med.*, **137**, 1711.

PETO, R., PIKE, M.C. & ARMITAGE, P. (1977). Design and analysis of randomized clinical trials requiring prolonged observations in each patient. *Br. J. Cancer*, **35**, 1.

SMALLEY, R.V., LEFANTE, J. & CARPENTER, J. (1983). A comparison of cyclophosphamide, adriamycin and 5-fluorouracil (CAF) and cyclophosphamide, methotrexate, 5-fluorouracil, vincristine and prednisolone (CMFVP) in patients with advanced breast cancer. *Breast Cancer Res. Treat.*, **3**, 209.

SMALLEY, R.V., MURPHY, S., HUGULEY, C.M. & BARTOLUCCI, A.A. (1976). Combination versus sequential five drug chemotherapy in metastatic carcinoma of the breast. *Cancer Res.*, **36**, 3911.

TORMEY, D.C., YELMAN, R., BAND, P.R. & 5 others (1982). Comparison of induction chemotherapies for metastatic breast cancer. *Cancer*, **50**, 1235.


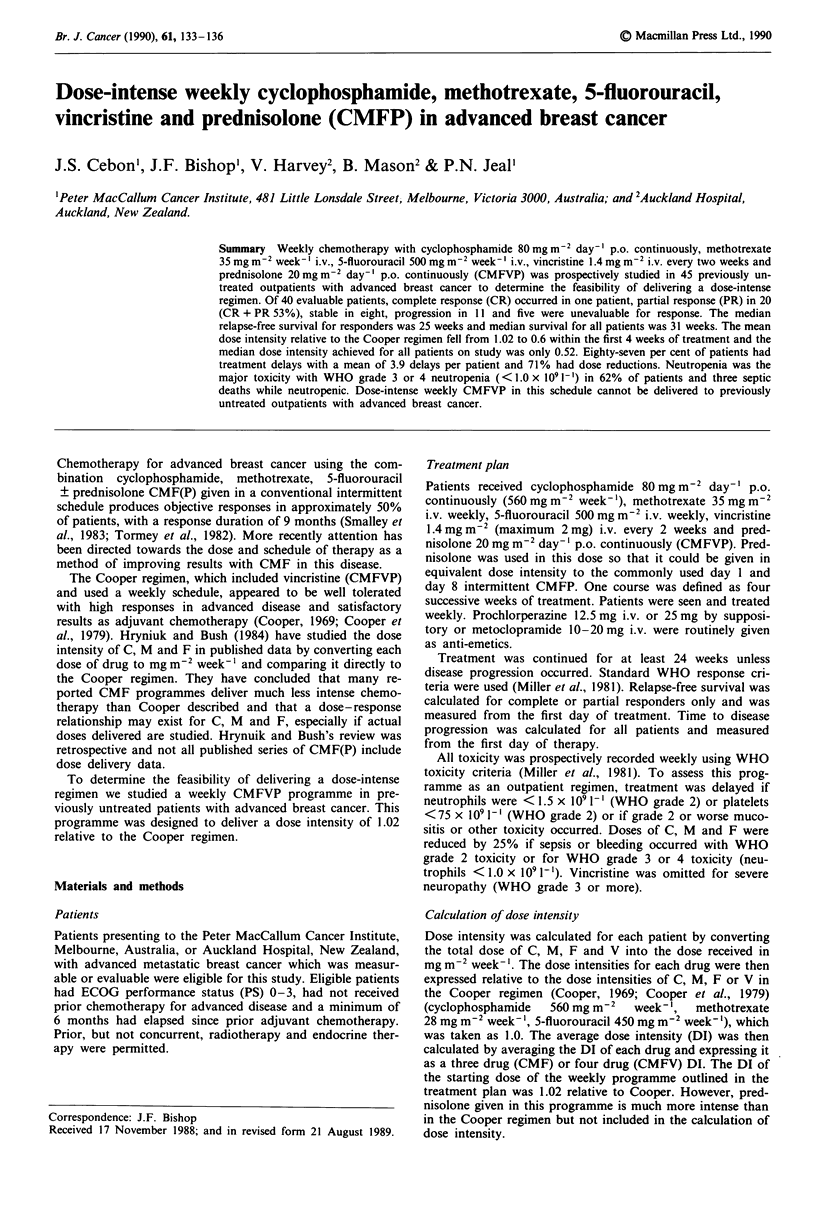

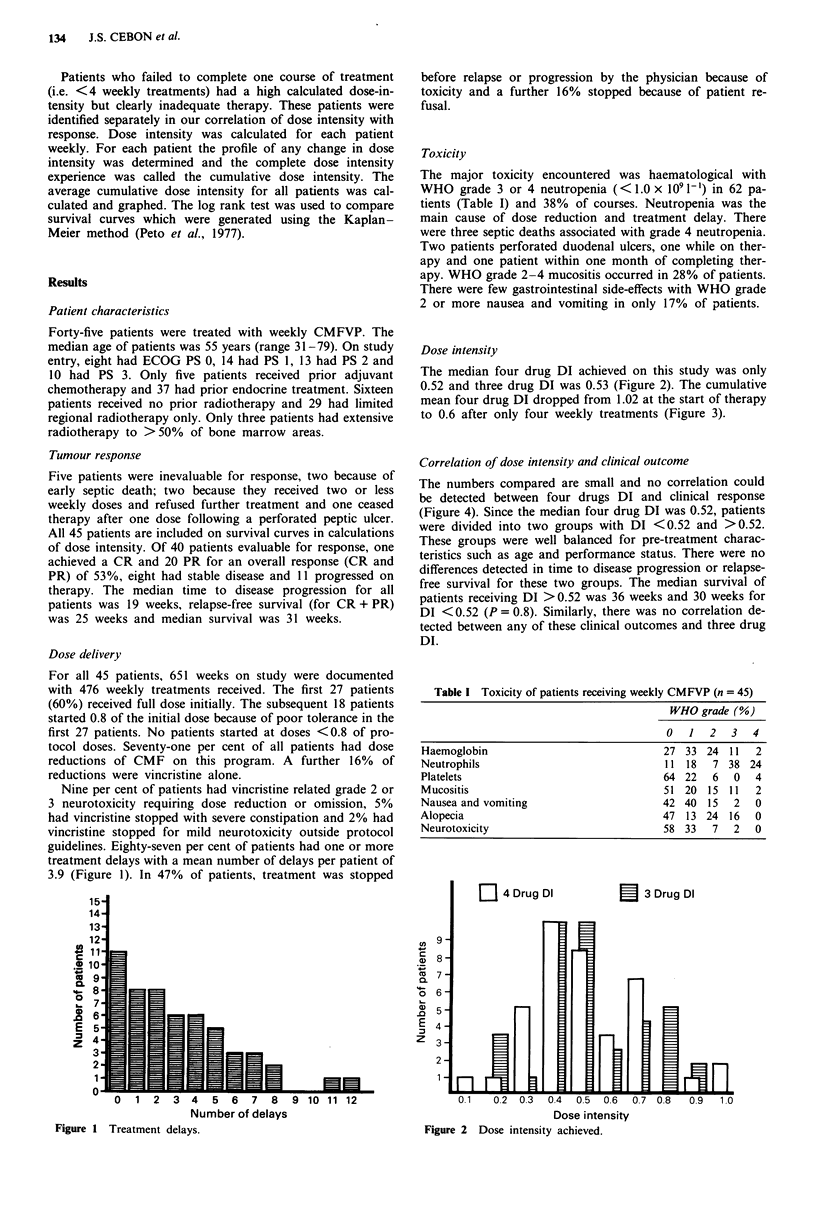

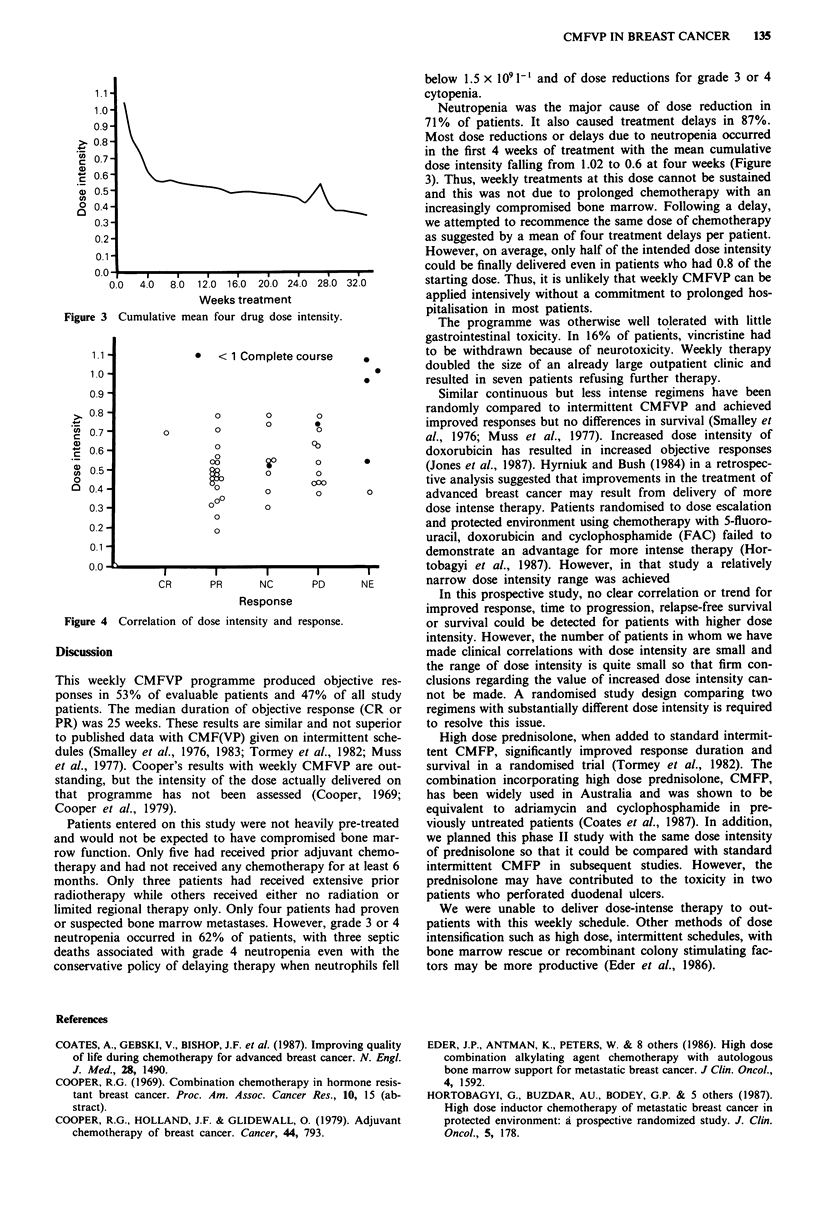

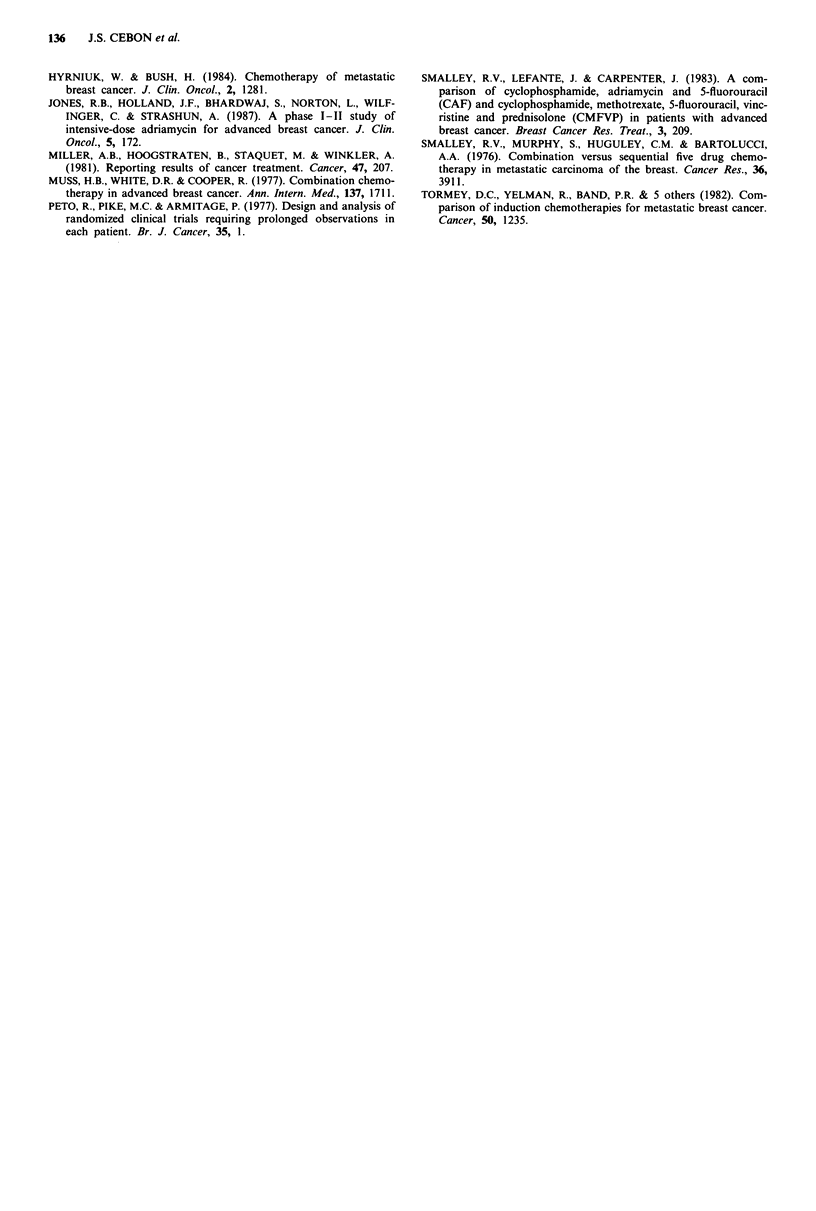


## References

[OCR_00461] Coates A., Gebski V., Bishop J. F., Jeal P. N., Woods R. L., Snyder R., Tattersall M. H., Byrne M., Harvey V., Gill G. (1987). Improving the quality of life during chemotherapy for advanced breast cancer. A comparison of intermittent and continuous treatment strategies.. N Engl J Med.

[OCR_00471] Cooper R. G., Holland J. F., Glidewell O. (1979). Adjuvant chemotherapy of breast cancer.. Cancer.

[OCR_00475] Eder J. P., Antman K., Peters W., Henner W. D., Elias A., Shea T., Schryber S., Andersen J., Come S., Schnipper L. (1986). High-dose combination alkylating agent chemotherapy with autologous bone marrow support for metastatic breast cancer.. J Clin Oncol.

[OCR_00481] Hortobagyi G. N., Buzdar A. U., Bodey G. P., Kau S., Rodriguez V., Legha S. S., Yap H. Y., Blumenschein G. R. (1987). High-dose induction chemotherapy of metastatic breast cancer in protected environment: a prospective randomized study.. J Clin Oncol.

[OCR_00489] Hryniuk W., Bush H. (1984). The importance of dose intensity in chemotherapy of metastatic breast cancer.. J Clin Oncol.

[OCR_00495] Jones R. B., Holland J. F., Bhardwaj S., Norton L., Wilfinger C., Strashun A. (1987). A phase I-II study of intensive-dose adriamycin for advanced breast cancer.. J Clin Oncol.

[OCR_00499] Miller A. B., Hoogstraten B., Staquet M., Winkler A. (1981). Reporting results of cancer treatment.. Cancer.

[OCR_00502] Muss H. B., White D. R., Cooper M. R., Richards F., Spurr C. L. (1977). Combination chemotherapy in advanced breast cancer.: a randomized trial comparing a three-vs a five-drug program.. Arch Intern Med.

[OCR_00505] Peto R., Pike M. C., Armitage P., Breslow N. E., Cox D. R., Howard S. V., Mantel N., McPherson K., Peto J., Smith P. G. (1977). Design and analysis of randomized clinical trials requiring prolonged observation of each patient. II. analysis and examples.. Br J Cancer.

[OCR_00510] Smalley R. V., Lefante J., Bartolucci A., Carpenter J., Vogel C., Krauss S. (1983). A comparison of cyclophosphamide, adriamycin, and 5-fluorouracil (CAF) and cyclophosphamide, methotrexate, 5-fluorouracil, vincristine, and prednisone (CMFVP) in patients with advanced breast cancer.. Breast Cancer Res Treat.

[OCR_00517] Smalley R. V., Murphy S., Huguley C. M., Bartolucci A. A. (1976). Combination versus sequential five-drug chemotherapy in metastatic carcinoma of the breast.. Cancer Res.

[OCR_00523] Tormey D. C., Gelman R., Band P. R., Sears M., Rosenthal S. N., DeWys W., Perlia C., Rice M. A. (1982). Comparison of induction chemotherapies for metastatic breast cancer. An Eastern Cooperative Oncology Group Trial.. Cancer.

